# Supplementary material for: A Nasal Vaccine Candidate, Containing Three Antigenic Regions from SARS-CoV-2, to Induce a Broader Response
Source: Vaccines (Basel). 2024 May 28;12(6):588. doi: 10.3390/vaccines12060588 (PMC11209543; doi:10.3390/vaccines12060588)
Supplement: Supplementary file 1 [file vaccines-12-00588-s001.zip › vaccines-3006852-supplementary.pdf]

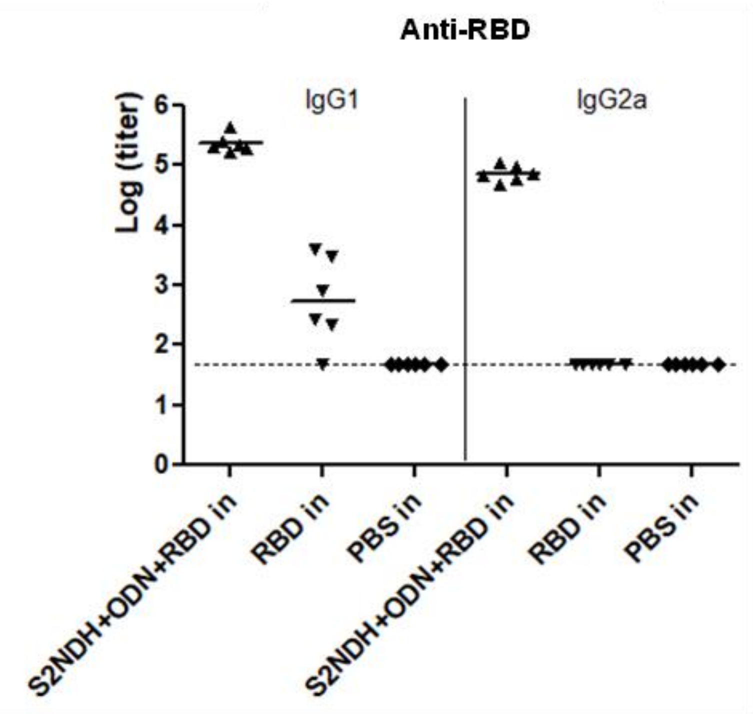

Figure S1. Anti-RBD IgG subclasses response generated by the intranasal administration of the bivalent formulation S2NDH+ODN-39M+RBD. Twenty-seven days after the third immunization, mice were sacrificed and IgG1, IgG2a antibody responses were evaluated in sera by ELISA.
